# A Parameter Study for 3D-Printing Organized Nanofibrous Collagen Scaffolds Using Direct-Write Electrospinning

**DOI:** 10.3390/ma12244131

**Published:** 2019-12-10

**Authors:** Frank A. Alexander, Lee Johnson, Krystaufeux Williams, Kyle Packer

**Affiliations:** 1 The Geneva Foundation, Tacoma, WA 98402, USA; 2 Meadowave LLC, NE Washington, DC 20012, USA; 3 Chemistry Division, Center for Corrosion Science and Engineering (CCSE), US Naval Research Laboratory, SW Washington, DC 20375, USA; krystaufeux.williams@nrl.navy.mil; 4 Womack Army Medical Center, Fort Bragg, NC 28310, USA; kyle.t.packer.mil@mail.mil

**Keywords:** electrospinning, collagen, direct-write electrospinning, scaffolds

## Abstract

Collagen-based scaffolds are gaining more prominence in the field of tissue engineering. However, readily available collagen scaffolds either lack the rigid structure (hydrogels) and/or the organization (biopapers) seen in many organ tissues, such as the cornea and meniscus. Direct-write electrospinning is a promising potential additive manufacturing technique for constructing highly ordered fibrous scaffolds for tissue engineering and foundational studies in cellular behavior, but requires specific process parameters (voltage, relative humidity, solvent) in order to produce organized structures depending on the polymer chosen. To date, no work has been done to optimize direct-write electrospinning parameters for use with pure collagen. In this work, a custom electrospinning 3D printer was constructed to derive optimal direct write electrospinning parameters (voltage, relative humidity and acetic acid concentrations) for pure collagen. A LabVIEW program was built to automate control of the print stage. Relative humidity and electrospinning current were monitored in real-time to determine the impact on fiber morphology. Fiber orientation was analyzed via a newly defined parameter (spin quality ratio (SQR)). Finally, tensile tests were performed on electrospun fibrous mats as a proof of concept.

## 1. Introduction

Electrospinning is a well-established technique for creating nanofibers that can be used for a variety of applications. The core technique was first demonstrated more than a century ago [1]; however, recent interest in biomaterials has caused a resurgent interest in it as a method for developing scaffolds for regenerative medicine and wound dressings [2]. In a classic far-field electrospinning (FFES) setup, a syringe pump perfuses a polymer solution through a conductive syringe tip to a grounded collector with a high voltage power supply. When enough voltage is applied between the syringe and collector circuit, charge buildup in the viscoelastic fluid pumped through the syringe deforms the meniscus into a “Taylor cone” and a nanofiber jet erupts from the tip of the cone when the electrical force overcomes surface tension. As the nanofiber jet is drawn closer to the surface, the carrier liquid evaporates and nanofibers form. Dried fibers accumulate onto the grounded collector electrode surface located below the syringe. 

Typically, in FFES the syringe tip is held a large distance (centimeters) from a target. This results in nanofibers collecting in random “pile of spaghetti”-like orientations. Alongside bioprinted hydrogels [3], unorganized “biopaper” scaffolds [4] have been popular due to their ease of production and ability to recapitulate biochemical cues seen in vivo by using natural polymers and a nanoporous structure. However, these systems still lack critical microarchitecture especially when creating tissue analogues of more ordered nanofibrous structures [5]. The lack of systematically organized fibrils may result in insufficient mechanical properties of these artificial tissues. Some groups have designed bioreactors to physically stimulate cellular production of organized collagen structures but production times can be extensive [6]. Leveraging additive manufacturing techniques to produce more similar tissue-specific analogues may increase utility.

Various groups have developed methods to finely pattern electrospun nanofibers into organized structures. Fibers have been directed onto spinning mandrel collectors to create fiber spools [7], micropatterned via insulator-collector geometries to induce specific shapes [8] and positioned in precise locations by rapidly translating the collector with an X-Y stage [9,10,11,12]. The latter technique, sometimes termed direct-write electrospinning (DWES) is a relatively new additive manufacturing approach to electrospinning into highly organized confirmations. 

DWES significantly lowers the required voltage to induce Taylor cone formation by decreasing the syringe tip to collector distance and incorporates a translating X-Y stage to pattern collected nanofibers. Li et al. successfully lowered spinning voltages to thresholds as small as 50 V, enabling them to electrospin living bacteria immersed in a polyethylene oxide-water solution [10]. Furthermore, they demonstrated the ability to electrospin directly onto hydrogels and PDMS films as well as electrospin freestanding fibers across two spin “initiators.” [8]. Polymer melts have been elecrospun in melt electro-writing (MEW) to create organized fibrous scaffolds, but they are incompatible with heat sensitive polymers like collagen [13,14,15,16,17,18]. Additionally, varying polymer solutions and custom equipment make it is necessary to re-optimize methods across laboratories. This drawback is the primary disadvantage preventing DWES from being a scalable method for scaffold manufacturing.

Natural, synthetic, and even blended polymer biomaterials [2] have been employed in electrospinning to generate nanofiber constructs that imitate native extracellular matrix (ECM) for tissue regeneration [2]. Collagen is particularly interesting as a scaffolding material because it is highly abundant within mammalian ECM. Of the 29 types of native collagen, Type I makes up 70–90% of collagen within the human body and can be found in tightly organized fiber networks in tissues like the meniscus [19,20]. Despite this, instead of electrospinning pure collagen into organized nanofibrous collagen scaffolds, the majority of researchers have opted for hydrogels or nanofibrous scaffolds with random orientation. These scaffolds are suitable in many applications, however, in many tissues precise fiber organization results in unique in vivo properties like transparency in the cornea [21]. For this reason, DWES is a superior method for producing organized collagen scaffolds.

Despite the extensive work to optimize parameters for traditional collagen electrospinning, organized collagen scaffolds have yet to be produced via DWES. Leveraging DWES as a 3D printing approach can help to further develop more complex and biomimetic scaffolds that mimic the native environment of cells or introduce bio-bandages that accelerate wound healing. A critical and often overlooked step in electrospinning is determining optimal environmental parameters for nanofiber production, as process temperature and relative humidity can directly impact fiber morphology. For this reason, it is critical to optimize a consistent production environment for repeatable fiber making in order to scale for large-scale manufacturing of scaffolds. Many prior groups have done exhaustive studies on solution characteristics such as solvent composition [22] but relatively few have done exhaustive studies on environmental parameters. To our knowledge, no such studies on environmental parameters have been performed for direct-write electrospinning of pure collagen.

Here, we present a systematic approach to optimizing critical parameters for DWES of collagen employing an environmental chamber for strict monitoring of relative humidity during the electrospinning process. Our printer, capable of manufacturing highly ordered 3D printed scaffolds from nanofibrous collagen, was evaluated by optimizing electrospinning characteristics of collagen across an array of varied process parameters including spin voltage, relative humidity and acetic acid concentration. The print procedure was automated using custom LabVIEW software built to control translation of the print stage and import common G-code files. Additionally, process parameters were monitored in real-time to assess the impacts of small-scale voltage and humidity fluctuations effect fiber morphology and placement. Electrospun collagen fibers were examined to ascertain the morphology of nanofibers produced and the quality of fiber placement which we have defined as the spin quality ratio (SQR). Finally, to demonstrate their mechanical properties we have tensile tested the manufactured nanofibrous fiber mats.

## 2. Materials and Methods

### 2.1. Electrospinning Printer Setup

**Figure 1:**
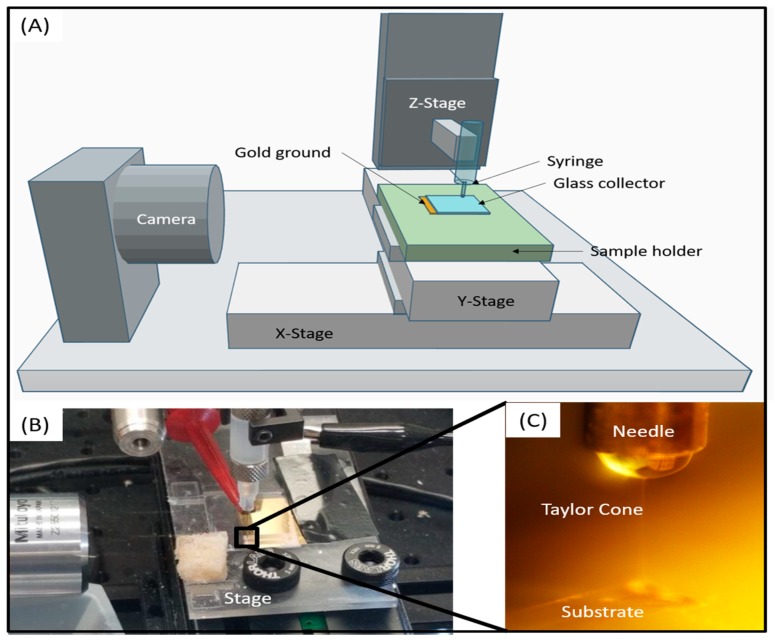
(**A**) A schematic depiction of the complete electrospinning printer setup. (**B**) The direct-write printhead and collector stage. (**C**) Detail image showing single fiber erupting from Taylor cone on surface of the droplet meniscus and depositing on substrate collector.

An electrospinning 3D printer was designed for all collagen fiber production experiments. The electrospinning printer contains four main components: an X-Y stage, a syringe pump, a high voltage DC power supply and a Z-positioner. Two DC servo linear translation stages (ThorLabs, Newton, NJ, USA) were bolted together to serve as the main X-Y positioner. Attached to the X-Y positioner, a custom stage (3D printed with a Form2 from FormLabs, Somerville, MA, USA) houses an integrated ground plane made from a gold-coated glass slide. The ground plane is connected to a metal microscope slide holder that is clipped to the negative terminal of a PS300 high voltage power supply (Stanford Research Systems, Sunnyvale, CA, USA). An NE-1000 syringe pump (New Era, Farmingdale, NY, USA) was used to drive collagen solution from a 3 mL syringe to a 27-gauge needle tip mounted to a third screw-driven translation stage for Z-positioning. A National Instruments PXIe-1071 workstation with a custom LabVIEW program was used to synchronize the voltage supply, syringe pump and stage into a coordinated print routine. Within the program, G-code can be uploaded to print complex geometries. The entire print setup is housed in an ETS-5532 environmental chamber to control temperature and humidity (Figure 1). 

### 2.2. Collagen Solution Formula

Collagen solutions were made with acetic acid and evaluated for their ability to form aligned fibers during the electrospinning process. 500 mg of type I bovine dermis derived atelocollagen powder (Cosmo Bio USA, Carlsbad, CA) was vortexed in 2 mL of 40, 50 and 60% acetic acid (Sigma-Aldrich, St. Louise, MO, USA) diluted in deionized water until completely dissolved (approximately 30 min) to a concentration of 250 mg/mL. The solution was then preloaded into a 3 mL syringe and placed onto the syringe pump. From there, it was coupled to the dispensing needle using 24 Gauge polytetrafluoroethylene (PTFE) tubing with luer lock hubs from Hamilton (Reno, NV, USA). 

### 2.3. Electrospinning Process

Collagen fibers were electrospun directly onto glass slides pre-treated with dry film Teflon spray (Dupont, Midland, MI, USA). Up to 600 lines of collagen were spun onto samples 30 lines at a time with a pitch of 15 microns and an approximate syringe tip to substrate distance of ~0.580 mm. A two-step voltage application was employed where 1600 V was initially applied to induce fiber formation and the voltage was then lowered to a preset maintenance voltage after three seconds and held there for the duration of the run. Temperature, relative humidity, and maintenance voltage were all controlled via the custom LabVIEW software and parameters were varied to determine optimal spin parameters. Table 1 shows the experimental parameters tested. Optimal spin quality was determined by counting the number of organized, straight fibers drawn on the sample using image processing in ImageJ and Matlab.

### 2.4. Scanning Electron Microscopy (SEM)

Electrospun collagen fibers were examined with scanning electron microscopy to ensure a fibrous morphology and to compare microstructural differences across multiple spinning parameters. Samples to be imaged were sputter coated with a thin layer of carbon (~30 nm) prior to loading in the SEM. Scanning electron microscopy images were acquired using a Hitachi SU-70 SEM (Tokyo, Japan). All electron microscopy was performed at the Advanced Imaging and Microscopy Laboratory (AIMLab) at the University of Maryland Nanocenter.

### 2.5. Definition of High-Quality Fibers

**Figure 2:**
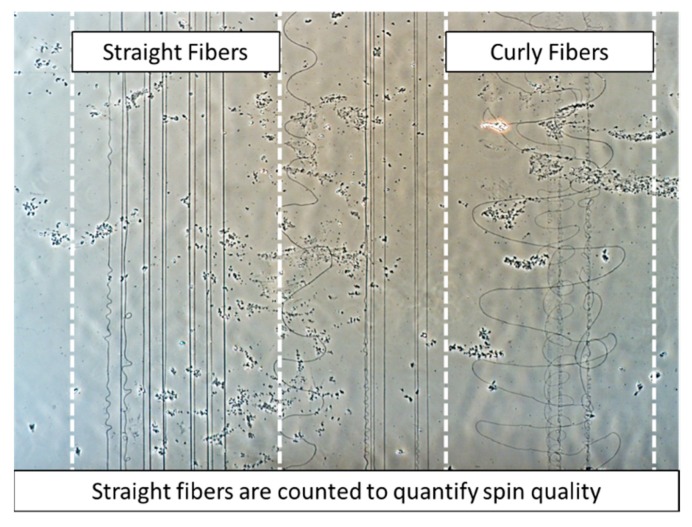
A light microscopy image comparing straight fibers and curly fibers produced during the electrospinning process. Straight fibers were detected using an image processing algorithm and counted and curly fibers were filtered from counts.

The quality of fibers produced can vary drastically given the combination of chamber humidity, spin voltage, and acetic acid concentration. For this reason, a quantitative measure of spin quality was defined after preliminary investigations of direct-write electrospinning parameters. During each electrospinning experiment, a target number of straight collagen fibers was recorded along with the resultant number of straight fibers produced. Primarily, our use of direct-write electrospinning is as an additive manufacturing technique capable of producing highly organized electrospun scaffolds. As such, production of wavy or curly fibers reduce placement accuracy and predictability of the print technique, and hence, these fibers were excluded from the line counts (Figure 2). Here, we define the spin quality ratio (SQR) as Equation (1):

SQR = (# of straight lines produced)/(# of lines attempted),(1)

The SQR can be used as a plotted metric to estimate quantitatively the linearity of lines produced during the electrospin process. 

### 2.6. Fiber Counting Algorithm

**Figure 3:**
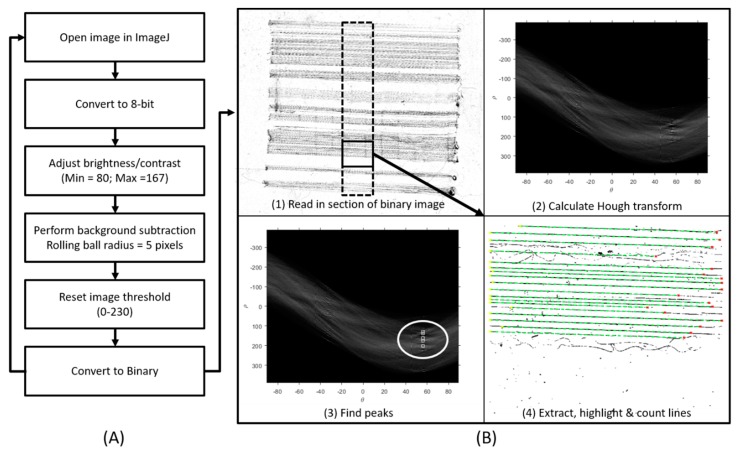
Block diagrams of (**A**) an image pre-processing algorithm for binarizing composite images of electrospun fibers and (**B**) a nanofiber counting algorithm that quantifies the number of straight fibers crossing the center of the imaged sample. Manual counting was performed to validate automated counts.

Samples were imaged with a Nikon Eclipse Upright microscope (Tokyo, Japan) with a motorized X-Y stage and a composite image of the entire slide was constructed using Nikon Elements BR (Basic Research) software (version 4.13.04, Tokyo, Japan). An ImageJ pre-processing macro was created to enhance the contrast between the background and fibers in order to binarize images. A Matlab algorithm was formulated to count the number of high-quality fibers deposited onto sample slides. The block diagram in Figure 3 outlines the procedures for (a) pre-processing images and (b) fiber calculations. First a single slide image was converted into an 8-bit format within ImageJ. Brightness and contrast were adjusted to 80 and 167, respectively. A rolling ball image background subtraction was performed to improve contrast further (rolling ball = 5 light disable) and the image threshold was set to 230. Finally, the image was exported as a binary image file into Matlab. 

In Matlab, a Hough Transform routine was used to approximate the number of organized horizontal fibers in the image. A 1000 × 1000-pixel subsection was first extracted along the centerline of the parent image and the Hough transform was calculated for that sub-image. Peaks were found within the transform image and used to identify horizontal lines within a given angular threshold. The entire sequence was repeated for the entire parent image. The algorithm was refined and validated via manual counts across several sample images.

### 2.7. Tensile Testing Procedures

To showcase our ability to build collagen fiber constructs, 30 layers of fibers were electrospun (1600 V, 30% RH, and 28 °C) with 15 µm spacing into 2.1 mm × 1.8 mm fiber mats and tensile tested. For comparison, fibers mats were also spun from a solution of hexafluoro-2-propranol (HFIP), another solvent commonly used for collagen fiber spinning [22]. HFIP-collagen solutions were mixed to a concentration of 8% *w*/*w* (138 mg/mL) and electrospun with the same parameters described above. Fiber mats were removed from Teflon coated glass substrates with a razor blade and the thickness of samples was measured before loading into an Electrodynamic Testing System from Test Resources (Shakopee, MN, USA). Samples were secured between two pieces of P400 sandpaper (3M, Maplewood, MN, USA) and stretched at a rate of 1.0 mm/minute until failure to determine ultimate tensile strength and Young’s modulus. Analysis of the results was performed using Monotonic software (Test Resources) and plotted with Excel. 

## 3. Results

### 3.1. Initial Characterization of Electrospun Collagen Fibers

**Figure 4:**
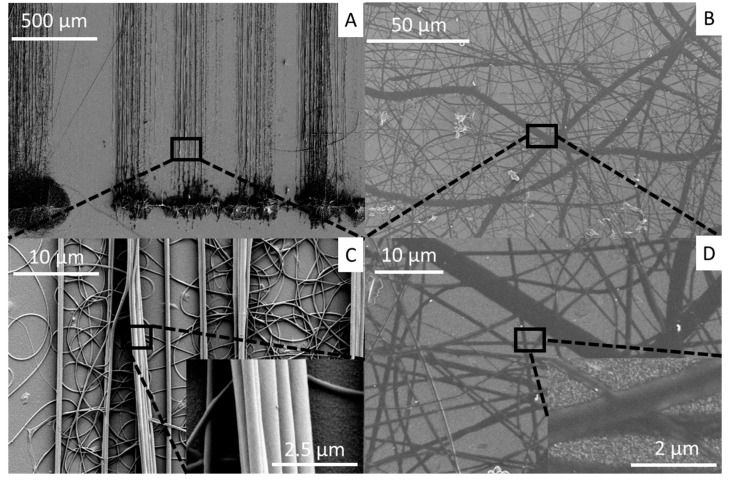
Scanning electron microscopy image of electrospun collagen nanofibers. Example image of electrospun fibers with good cylindrical morphology and fiber alignment at low (**A**) and high magnification (**C**). (Inset C) Fibers appear three dimensional and are tightly packed into stacks. Example image of electrospun fibers with poor morphology and random orientation at low (**B**) and high magnification (**D**). (Inset D) Fibers appear with a flat morphology and randomly arranged on the collector substrate.

Collagen fibers were spun directly onto Teflon-coated glass slides to evaluate spin quality for a given set of critical electrospinning parameters. Voltage, relative humidity, and acetic acid concentration were varied to establish optimal parameters for direct-write electrospinning as an additive manufacturing method. 30% acetic acid solution were also prepared and electrospun but was not spinnable at relative humidity lower than 50% (results not shown). SEM images of fibers produced with 40% acetic acid display most fibers are light in color, with clear shading. This hints at fibers being solid and cylindrical (Figure 4A,C). Figure 4B shows low quality fibers electrospun with suboptimal parameters. Fibers here appear uniformly dark in color with no shading appearing on the background. Closer inspection at higher magnifications (inset Figure 4D) reveal fibers seem to have a flat and planar morphology. Here, it is shown that fiber morphology can vary during the electrospinning process. Organized fibers appear in linear bundles of vertical lines with minimal distortion while unorganized fibers appear in loops that crowd the base of the substrate. 

### 3.2. Optimization of Collagen Electrospinning Parameters Based on SQR

**Figure 5:**
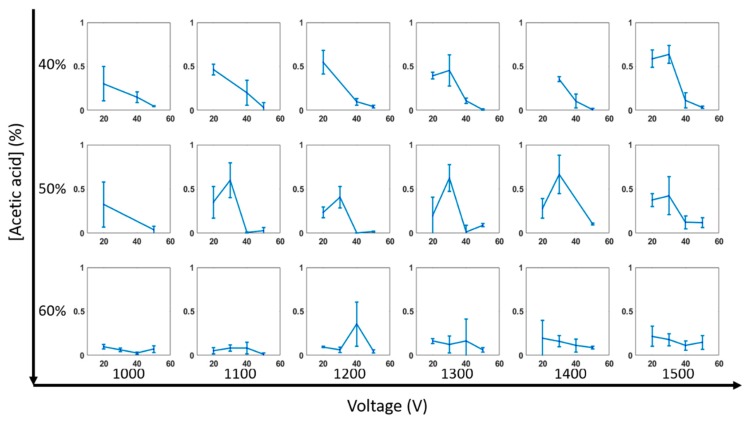
Data plot summary for relative humidity (RH) vs. spin quality ratio (SQR). The horizontal axes show data electrospun with increasing voltage and the vertical axes show data electrospun with increasing acetic acid concentration.

Composite images of electrospun samples were analyzed with a Matlab image processing sequence to detect fiber linearity. Spin quality ratio (SQR) was defined to quantitatively determine the organization of fibers produced via direct-write electrospinning. Figure 5 shows the resulting data set in a series of plots of RH vs SQR for all acetic acid concentrations (vertical axis) and voltages (horizontal axes). Generally, as RH increased, SQR decreased for all data sets, resulting in an increase of disorganized fiber spinning and a decrease of fiber linearity. 40% and 50% (1st and 2nd rows) acetic acid produced fibers with the highest SQRs, peaking at 0.66 and 0.63. Fibers produced with these conditions showed strong linearity when examined under light microscopy. High SQR indicates well-controlled fiber formation with significant linearity making these two concentrations preferable for controlled electrospinning. 

Further inspection of fiber spinning with 40% acetic acid shows that a lower relative humidity produces fibers with the best linearity peaking at 20 and 30%. Notably, as the voltage reaches 1300 and 1500 the SQR peaks at 30% RH for 40% acetic acid. For 50% acetic acid, a more consistent trend of peak SQR is found at 30% RH. 60% acetic acid consistently produced random and unorganized fibers with lower SQR values that rarely surpassed 0.3. Interestingly, for 50% relative humidity the calculated SQR is comparable for lower acetic acid concentrations. Generally, once the acetic acid concentration reaches 60%, SQR decreases significantly and fiber formation becomes more chaotic and less controllable.

For 40% acetic acid, as the voltage increases there is a slight increase in SQR, particularly at a relative humidity of 30%, while no apparent trends seem to be present for 50 and 60% concentrations. Microscopic inspection of samples (not shown) showed an increased presence of electrospun material at higher humidity. As the RH increases, the fiber production rate also increases, resulting in fibers piling up in an organized fashion as they exceed the XY motor translation speed. 

**Figure 6:**
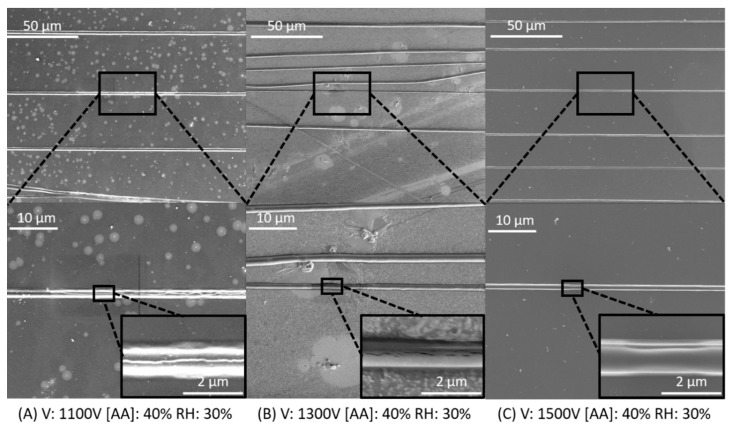
Scanning electron microscopy images of collagen nanofibers. Fibers were electrospun from 40% acetic acid at 30% relative humidity with varying voltages of (**A**) 1100 V, (**B**)1300 V, and (**C**) 1500 V. Fibers all have a clear cylindrical morphology and appear to have a high degree of fiber alignment on the substrate.

While light microscopy readily identifies fiber linearity, SEM images were recorded in order to closely examine fiber morphology as the voltage and relative humidity was manipulated. Figure 6 illustrates the morphology for collagen fibers produced with 40% acetic acid solution and 30% RH over voltages covering the entire investigated range. Fibers were solid with cylindrical morphology and high aspect ratio across the range of attempted voltages. Collagen fiber diameter remained consistent, measuring ~1–2 microns across the voltage range. Small cracks and surface defects seen on images can be attributed to defects in the carbon coating used for SEM inspection. These fibers exhibited both good morphology and high SQR, demonstrating highly organized fiber placement.

**Figure 7:**
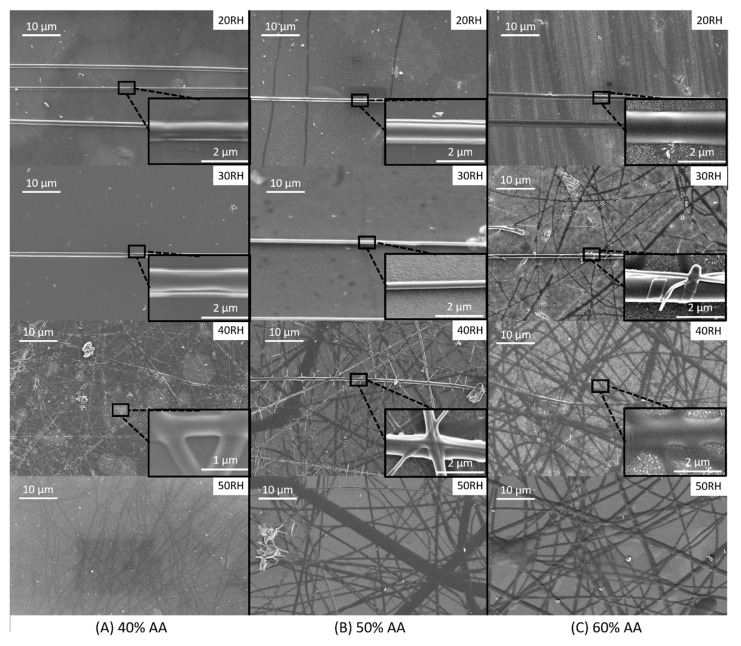
SEM images of fibers electrospun at 1500 V for various relative humidity (shown in upper right of each image) and acetic acid concentrations of (**A**) 40%, (**B**) 50%, and (**C**) 60%.

SEM images of samples electrospun at 1500 V across all investigated humidities and acetic acid concentrations are shown in Figure 7 in a more expansive study of fiber morphology and organization. At the lowest humidity (20 RH) collagen fiber morphology generally appeared solid, with few flat and planar fibers present on the surface. Fibers were well-organized, consistent with the SQR values found previously. 30 RH revealed similar morphology and arrangement for fibers formed from 40 and 50% acetic acid solutions, while 60% acetic acid solutions produced a combination of solid and flat fibers. Additionally, there is a significant increase in the volume of unorganized fibers. 

Once the relative humidity exceeded 30%, fiber morphology and organization degraded significantly across all acetic acid concentrations. At 40 RH, as the acetic acid concentration increased fibers became flatter in morphology. At the highest tested relative humidity, all fibers viewed took on a planar morphology. Under high magnification fibers appeared almost indistinguishable with the background and are distributed randomly across the sample space. This finding is consistent with the steep drop-offs in SQR found in the previous section.

### 3.3. Peak Loadand Young’s Modulus of Uncrosslinked Fibers

**Figure 8:**
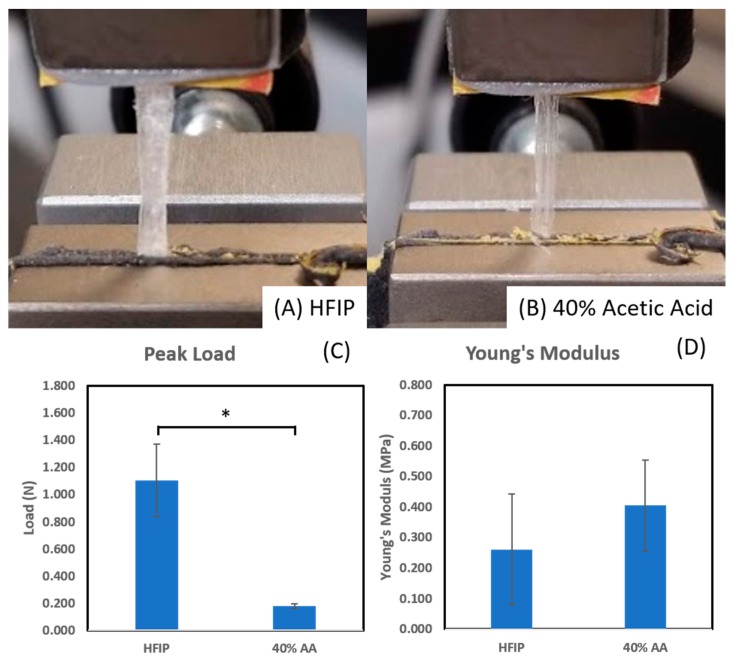
Example image of tensile testing system with collagen fiber mats made with (**A**) HFIP and (**B**) 40% acetic acid. Collagen fiber mats were stretched in the above configuration until failure and the (**C**) peak load (n = 4, *p* = 0.006) and (**D**) young’s modulus (n = 4, *p* = 0.268) were recorded for samples and the averages and standard deviations were plotted.

As a proof of concept, thick collagen nanofiber mats were electrospun at 30% relative humidity from 40% acetic acid (250 mg/mL collagen) and HFIP solutions (138 mg/mL collagen) to form 30 layers of fibers. These nanofiber mats were mechanically tested to measure ultimate tensile strength and Young’s modulus. Fiber mats were electrospun to a thickness of 30 layers. Figure 8A,B show the resultant fiber mat loaded into the tensile testing setup at the beginning of an experiment. It is apparent from the image that fiber mats spun from HFIP solution are thicker than mats from acetic acid solution. This is consistent with previous work showing electrospun collagen from HFIP solutions having larger fiber diameters than acetic acid solutions [23,24,25]. Figure 8C,D compare the average peak load and young’s modulus with standard deviation. Although the peak load for HFIP-based fiber mats is significantly higher (*p* = 0.006) than AA-based fiber mats, the young’s modulus show no statistically significant difference (*p* = 0.268). Future work and studies will look at further categorizing the influence of crosslinking, layer amount, and spinning solvent on material properties.

## 4. Discussion

### Relative Humidity, Acetic Acid Concentration and Fiber Morphology

Direct-write electrospinning exhibits potential as a method to produce nanofibrous scaffolds. Precise control over fiber placement in the future will allow researchers to build precision matrix scaffolds much like native ECM. Furthermore, bioanalogs of ordered tissue like cornea and meniscus can be produced for regenerative medicine and transplantation [26,27]. To effectively incorporate DWES as a 3D printing modality, environmental and process settings must be optimized for reproducibility. Relative humidity is a critical environmental parameter that contributes to systematically electrospinning fibers into an ordered structure. In addition, selecting the correct solvent is paramount to maintaining high quality electrospinning. For this reason, an exhaustive study was performed to extract the optimal relative humidity and acetic acid concentration for direct-write electrospinning of collagen nanofibers.

Based on the presented study, 40% acetic acid was confirmed to be the best solvent concentration for electrospinning collagen fibers. Pure acetic acid is well-known for its ability to solubilize collagen but initially was unsuitable as a solvent for electrospinning due to its high affinity to collagen and slow evaporation rate [28,29,30]. Early demonstrations of collagen electrospinning utilized hexafluoroisopropanol (HFIP) or trifluro-ethanol (TFE) as solvents to achieve a spinnable viscosity, but high toxicity of these fluoro-organic solvents make them unsuitable for everyday use [31]. Additionally, there are conflicting reports on how similar electrospun collagen produced from these solvents is to native collagen on a molecular level [32]. 

Acetic acid still shows promise as a solvent because it does not completely denature collagen in solution like HFIP and TFE [22]. Here, we analyzed the impact of acetic acid concentration on fiber morphology and organization. The highest concentration tested (60%) resulted in consistently poor fiber morphology and low control on fiber placement across all relative humidities. The lower concentrations (40 and 50%) resulted in solid, cylindrical fiber morphology and improved fiber placement, especially at relative humidities below 40%. From this we can deduce that diluting the acetic acid concentration with water increases the evaporation rate of the carrier solvent when the humidity is sufficiently low allowing fibers to dry prior to deposition on the collector. 

Evaporation of a solution is regulated by the temperature and humidity of the surrounding environment as well as the vapor pressure of the solution. Because the vapor pressure of water and acetic acid are very similar (2.5 kPa and 3.76 kPa, at 28 °C respectively) [33] the relative humidity seems to highly impact the rate of spinning and fiber morphology. Liu et al. demonstrated a diluted 40% acetic acid solution as a suitable electrospinning solvent for type I collagen, however relative humidity and other acetic acid concentrations were left unreported. Polk et al. reported dissolving collagen in acetic acid solutions of 20–50% for compressed air-driven pneumatospinning but do not report environmental parameters or results for solutions other than 40% [25]. Despite its critical role in determining fiber morphology, few researchers have reported on the relative humidity required to produce electrospun collagen. This may be due to the prevalence of fluoro-alcohol use as a solvent. Our work fills a void in the literature showing the relationship between collagen electrospinning solvent, relative humidity and fiber morphology and organization.

## 5. Conclusions

In summary, an exhaustive study was performed to ascertain ideal acetic acid concentration, relative humidity and voltage parameters for direct-write electrospinning of highly organized collagen nanofibers for scaffold production. A custom electrospinning printer was designed and built in order to produce organized single lines of collagen nanofibers with cylindrical morphology. Acetic acid concentration, relative humidity and spin voltage were all varied to establish best practices for producing single fibers of collagen with high linearity and control of fiber placement. 

An acetic acid concentration of 60% produced wet fibers that absorbed onto the substrate with an increasingly planar morphology with increasing humidity, despite showing a degree of control over fiber placement for 20% relative humidity. Acetic acid concentrations of 40 and 50% were found to consistently produce solid fibers with cylindrical morphology at relative humidity lower than 40% making them optimal concentrations for direct-write electrospinning of collagen. As a rule of thumb, the relative humidity should be kept ≤30% in order to maintain a solid fiber morphology. Additionally, SQR most consistently peaked at 20% and 30% relative humidity for 40% and 50% acetic acid, respectively. Finally, the mechanical properties of fiber mats electrospun with 40% acetic acid and HFIP as a solvent were tested to show the ability to electrospin large scaffolds of materials. The process parameters identified in this work can enable researchers to direct-write electrospin pure collagen scaffolds with specific morphologies that more accurately mimic native ECM in specific tissues.

## Figures and Tables

**Table 1:** The list of parameters surveyed for optimizing collagen nanofiber formation.

Parameters						
[Acetic Acid] (%)	40	50	60	-	-	-
Relative Humidity (%)	20	30	40	50	-	-
Voltage (V)	1000	1100	1200	1300	1400	1500
